# The role of proinflammatory response and the kynurenine pathway in the association between childhood maltreatment and lifetime substance use disorder

**DOI:** 10.1016/j.ynstr.2026.100820

**Published:** 2026-04-21

**Authors:** Lars Östman, Elisabeth R. Paul, He Zhang, Evelina Larsson, Irene Perini, Lilly Schwieler, Sophie Erhardt, Lovisa Holm, Åsa Axén, Leah M. Mayo, Markus Heilig, Andrea J. Capusan

**Affiliations:** a Center for Social and Affective Neuroscience, Department of Biomedical and Clinical Sciences, Linköping University, Linköping, Sweden; b Department of Psychiatry, Linköping, Department of Biomedical and Clinical Sciences, Linköping University, Linköping, Sweden; c Department of Physiology and Pharmacology, Karolinska Institute, Stockholm, Sweden; d Department of Psychiatry, Mathison Centre for Mental Health Research and Education, Hotchkiss Brain Institute, University of Calgary, Calgary, Canada

## Abstract

**Introduction:**

Childhood maltreatment (CM) is a risk factor for adult psychiatric and substance use disorders (SUD). Retrospectively assessed CM has been linked to increased proinflammatory cytokines, including IL-6. Induced by inflammation, the neurotoxic branch of the kynurenine pathway has been implicated in psychiatric disorders and SUD. This study explored proinflammatory responses and kynurenine metabolites following acute stress in participants with, and without, prospectively recorded CM, with or without, lifetime SUD.

**Methods:**

The study included 89 participants, divided into 4 groups based on the presence or absence of prospectively assessed CM and lifetime SUD: CM + SUD, n = 24, CM only, n = 20, SUD only, n = 22, and healthy controls (HC), n = 23. Participants underwent an acute stress task. Blood was collected at five time-points measuring IL-6 and kynurenine metabolites. Linear mixed models assessed the effects of CM, SUD, and time on IL-6 and kynurenine metabolite levels.

**Results:**

Participants with prospectively recorded CM had higher baseline IL-6 compared to those without CM (mean difference = 0.37, 95% CI = 0.09-0.57, p = 0.008). Stress increased IL-6 in all participants (p < 0.001), with no significant group differences. We found no association between CM exposure and KYNA or QUIN concentrations. Participants with SUD, irrespective of CM-status, had a lower KYNA/QUIN ratio (mean difference: 0.02, 95% CI: 0.00-0.04, p = 0.047).

**Discussion:**

Our findings of low-grade proinflammatory activity support the hypothesis that CM contributes to long-term immune system alterations, but these findings do not support the role of the kynurenine pathway in this process. However, increased neurotoxicity through kynurenine metabolism was associated to SUD-diagnosis.

## Highlights

• Documented exposure to childhood trauma increases inflammatory markers.
• The ratio of kynurenic acid to quinolinic acid is decreased in individuals with SUD.
• Inflammatory markers increase over time, after an acute laboratory stressor.
• Childhood trauma is not associated with changes in kynurenine metabolism.

## 1 Introduction

Childhood maltreatment (CM), including physical, sexual and emotional abuse, and physical and emotional neglect (Mullen et al., 1996) is an established risk factor for psychiatric and substance use disorders in adulthood (Daníelsdóttir et al., 2024; Grummitt et al., 2024; Huang et al., 2012), yet the biological mechanisms which link early adversity to long-term psychiatric or substance related outcomes remain incompletely understood.

One proposed pathway involves persistent alterations in the innate immune system (A. Danese and Lewis, 2017). Exposure to CM during sensitive developmental periods may induce long-lasting immune dysregulation, resulting in chronic low-grade inflammation in adulthood (Georgountzou and Papadopoulos, 2017; Andrea Danese and J Lewis, 2017). This proinflammatory state may arise through sustained activation of the sympathetic nervous system (Mondelli and Vernon, 2019; Elenkov et al., 2000), leading to increased circulating immune cells and enhanced production of inflammatory cytokines (Schorr and Arnason, 1999). Consistent with this hypothesis, a meta-analysis of studies using retrospectively reported CM found elevated levels of proinflammatory cytokines, including interleukin-6 (IL-6) (Baumeister et al., 2016). However, evidence from cohorts with prospectively documented CM exposure remains scarce, with only one study reporting increases in some—but not all—markers of inflammation (Bourassa et al., 2021). The lack of data on prospectively recorded CM exposure is problematic because of multiple sources of bias in retrospective recall (Colman et al., 2016; Fergusson et al., 2000) and the poor overlap between retrospectively recalled and prospectively documented CM (Reuben et al., 2016; Baldwin et al., 2019). Thus, data derived from prospectively assessed CM has the potential to reveal novel insights not otherwise detectable in samples relying solely on retrospective assessments of CM exposure.

Proinflammatory cytokines are sensitive not only to chronic stress exposure but also to acute psychosocial stress (Marsland et al., 2017; Eisenberger and Cole, 2012). Experimental studies consistently demonstrate that acute psychosocial stress increases circulating IL-6 levels in healthy individuals (Marsland et al., 2017). Whether early-life adversity alters this immune reactivity to acute stress remains largely unknown. Investigating acute stress responses may therefore provide insight into mechanisms through which CM shapes long-term vulnerability or resilience to stress-related disorders, including substance use disorders (SUD).

Inflammation may also influence neurobiological pathways implicated in psychiatric risk. Elevated IL-6 has been proposed to shift tryptophan metabolism toward the kynurenine pathway. In this pathway, tryptophan is metabolized to kynurenine and subsequently to several neuroactive metabolites (Paul et al., 2022), including quinolinic acid (QUIN), a neurotoxic NMDA receptor agonist, and kynurenic acid (KYNA), a neuroprotective NMDA receptor antagonist (Schwarcz et al., 2012; Stone and Perkins, 1981; Birch et al., 1988; Hertelendy et al., 2018). Higher IL-6 levels have been associated with increased QUIN and reduced KYNA concentrations (Schwieler et al., 2015; Ruddick et al., 2006; Guillemin, 2012). Alterations in this pathway have been implicated in SUD. A shift toward increased QUIN and decreased KYNA has been reported in SUD populations (Morales-Puerto et al., 2021). Both preclinical (Poeggeler et al., 1998) and clinical studies indicate lower KYNA levels associated with SUD (Wang et al., 2023). A clinical study also suggests increased QUIN in individuals with alcohol use disorder (Leclercq et al., 2021). Together, these findings suggest that kynurenine pathway metabolites may contribute to both vulnerability (e.g., increased QUIN) and resilience (e.g., higher KYNA) to SUD following CM.

Despite these potential mechanistic links, the relationship between CM and the kynurenine pathway remains poorly characterized. Animal models suggest that early-life stress alters kynurenine metabolism, but no human studies have systematically examined long-term effects of CM on kynurenine metabolites. Furthermore, research on kynurenine responses to acute psychosocial stress in humans is sparse and yields inconsistent findings (La Torre et al., 2021; Herhaus et al., 2021; Chiappelli et al., 2014). As a result, the effects of chronic and acute stress exposure on kynurenine pathway remain largely unknown.

To address these gaps, we examined baseline levels and stress-induced changes in IL-6 and kynurenine pathway metabolites from a unique cohort of young adults with prospectively documented CM exposure (Capusan et al., 2021; Perini et al., 2023). Participants with and without lifetime SUD were compared to unexposed controls with and without lifetime SUD. We further investigated the potential role of kynurenine metabolites in risk versus resilience to SUD by examining the KYNA/QUIN ratio across groups.

Experimental studies consistently demonstrate that acute psychosocial stress increases circulating IL-6 levels in healthy individuals (Marsland et al., 2017). Whether early-life adversity alters this immune reactivity to acute stress remains largely unknown. Investigating acute stress responses may therefore provide insight into mechanisms through which CM shapes long-term vulnerability or resilience to stress-related disorders, including substance use disorders (SUD).

Based on prior literature, our hypothesis was that participants exposed to CM would have increased baseline IL-6 levels and an increased IL-6-reactivity to acute stress exposure, accompanied by alterations in kynurenine metabolism. To test these hypotheses, IL-6 and kynurenine metabolites were measured at baseline and in response to a standardized psychosocial stressor in individuals with and without documented CM exposure, with and without life-time SUD.

## 2 Materials and methods

### 2.1 Study design

The study is part of a larger follow-up of young adults with prospectively documented severe CM and matched controls, as described in (Löfberg et al., 2023; Perini et al., 2023). The study was approved by the Regional Ethics Review Board in Linköping, Sweden (Dnr, 2015/256-31, and 2017/41-32).

### 2.2 Participants

Recruitment took place between March 2017 and July 2020. Using regional health records, we identified former patients from a specialized child and adolescent psychiatry trauma treatment unit, now young adults, 18 years or older (n = 470) (Perini et al., 2023; Löfberg et al., 2023).

From this cohort we then identified all individuals with both CM exposure, verified from the medical records and lifetime contact with SUD services (CM + SUD, n = 65). Individuals without confirmed CM, those who had emigrated, were deceased, lacked contact details, or had any current or lifetime bipolar or psychotic disorder, organic brain disorder, current suicidality, or cognitive impairment were excluded. Following the initial eligibility assessment, 8 met exclusion criteria, and 2 were deceased. The remaining 55 were invited to participate, and 33 were included.

Using the same regional register, for each CM + SUD participant, we identified sex- and age matched CM exposed participants with no lifetime SUD diagnosis or treatment contact (CM only). From 140 C M only subjects invited to participate in the study, 25 completed screening and inclusion.

For each CM + SUD participant, we also recruited sex- and age-matched clinical controls with lifetime SUD but no recorded CM exposure (SUD only). These clinical controls were recruited through the same medical records and through advertisements from addiction clinics in the region. Of 106 eligible for assessment, 25 SUD-only participants were included.

Sex- and age-matched healthy controls (HC) with no psychiatric disorder or CM were recruited by advertising on Facebook, among healthy participants in previous studies, and among students at Linköping University. From 34 invited 24 H C finished the study.

Of 107 included participants, 89 individuals completed laboratory testing, as follows: CM + SUD, n = 24, (50% female; median age = 28.5, IQR: 26.0-31.3); CM only, n = 20 (65% female; median age = 29.0, IQR: 25.0-32.3); SUD only, n = 22 (45% female; median age = 27.0, IQR: 26.0-29.0); and HC, n = 23 (52% female; median age: 27.0, IQR: 25.0-30.0). Those who completed laboratory testing only differed in sex (p = 0.02), but not in any other way (Table 1).

**Table 1:** **Study populations, overall and grouped over childhood maltreatment (CM) and lifetime substance use disorder (SUD) status.** AUDIT: alcohol use disorder identification test, a significant difference between participants with a history of, or ongoing, alcohol use disorder. DUDIT: drug use disorder identification test, a significant difference between participants with a history of, or ongoing, substance use disorder; CTQ: childhood trauma questionnaire, a significant difference between participants with a history of childhood maltreatment, or substance use disorder and healthy controls.

Characteristics	Overall, N = 89	CM-only N = 20^a^	CM + SUD N = 24^a^	SUD-only N = 22^a^	HC N = 23^a^	p-value^b^
**Age at testing** (years)^a^	28.0 (25.0. 31.0)	29.0 (25.0. 32.3)	28.5 (26.0. 31.3)	27.0 (26.0. 29.0)	27.0 (25.0. 30.0)	0.5
**Age at contact with CM treatment unit** (years)^a^	13.0 (8.0. 15.3)	12.0 (8.0. 14.0)	13.5 (9.5. 16.3)	NA	NA	0.2
**Psychotropic medication (%)**	30 (33%)	4 (20%)	12 (56%)	12 (56%)	2 (8%)	**0.003**
**Sex (Female)**	42 (47%)	7 (35%)	12 (50%)	12 (55%)	11 (48%)	0.6
**BMI**^a^	23.1 (21.1. 25.7)	21.8 (20.8. 25.2)	24.0 (20.8. 27.8)	24.9 (21.5. 26.8)	23.0 (21.4. 23.8)	0.4
**AUDIT**^a^	4.0 (2.0. 8.0)	3.0 (2.0. 5.3)	7.0 (4.8. 13.5)	4.0 (2.3. 9.0)	3.0 (2.0. 4.0)	**0.002**
**DUDIT**^a^	0.0 (0.0. 4.0)	0.0 (0.0. 0.0)	0.0 (0.0. 4.3)	4.0 (2.5. 7.8)	0.0 (0.0. 0.0)	**<0.001**
**CTQ**^a^	37 (29. 57)	54 (35. 71)	47 (37. 63)	41 (30. 48)	26 (26. 31)	**<0.001**

a Median (IQR); n (%).

b Derived from Kruskal-Wallis rank sum tests for continuous variables and Pearson's Chi-squared test for dichotomous variables.

### 2.3 Study procedure

After screening, eligible participants were invited to an in-person psychiatric assessment using the MINI-7.0.2 (Sheehan et al., 1998) based on DSM-5 (American Psychiatric Association, 2013). Participants also completed the childhood trauma questionnaire (CTQ) (Bernstein et al., 2003), Alcohol Use Disorder Identification Test (AUDIT) (Saunders et al., 1993) and the Drug Use Disorder Identification Test (DUDIT) (Berman et al., 2005). Controls were excluded if CM was found in medical records or emerged from the PTSD module in MINI-7.

In a second session, participants completed a series of behavioral tasks and laboratory tests designed to assess emotional and stress reactivity, including blood levels of cortisol and endocannabinoids as presented earlier (Perini et al., 2023). Data presented here were acquired during a task using the International Affective Picture System, IAPS, earlier described (Perini et al., 2023) and the Maastricht Acute Stress Test (MAST) (Smeets et al., 2012). The MAST is a 10-min task involving alternating hand immersion in ice-cold water and mental arithmetic trials with negative socio-evaluative feedback to assess acute stress reactivity. The MAST is a robust established stress paradigm, which combines features of the Trier Social Stress Test (Kirschbaum et al., 1993) and the Cold Pressor Test, CPT (Schwabe et al., 2008). The cortisol-response following the MAST is both significantly higher and of longer duration, compared to the response following the CPT, indicating a compound effect of physical and social stress on the cortisol response (Smeets et al., 2012). Participants were asked to assess their perceived subjective stress after the MAST-task, using a 100 mm visual analog scale, measuring 0-100.

### 2.4 Blood samples

Participants received an indwelling i.v. catheter and blood samples were then collected to assess baseline concentrations and stress-induced changes in peripheral proinflammatory markers and kynurenine metabolites. Samples were collected using EDTA BD vacutainer tubes via the indwelling catheter in the arm not submerged in the water, at the following five timepoints: 15 min prior to MAST stress test, (T-15), immediately prior to MAST test (T0), immediately following stress exposure (T15), and twice during the recovery period (T30 and T45).

Samples were immediately centrifuged for 10 min at 4 °C, 2000g, aliquoted, and stored at −20 °C. The samples were later stored at −70 °C, until analyses were conducted.

### 2.5 IL-6 analysis

The V-PLEX proinflammatory Panel 1, from the MSD Multi-spot assay system was used to determine IL-6 concentrations in blood. According to manufacturer instructions (Meso Scale DiagnosticsLLC, 2024), the frozen samples were thawed and added to a plate provided by the manufacturer together with a solution containing detection antibodies for IL-6, with electrochemiluminescent (ECL) labels, MSD SULFO-TAG, and an analyte which binds to IL-6. Once the IL-6-analyte-detection antibody-complex was formed, a buffer was added which created the appropriate chemical environment for ECL and a voltage was applied to the solution which caused the ECL-labels to emit light. By measuring the intensity of the emitted light, a quantitative measure of each analyte was obtained, which corresponds to the level of IL-6 in the sample. This method has been validated for reliable quantification of IL-6 -concentrations in the range 0.63-49 pg/mL (Lee et al., 2006). The limit of detection for the analysis was 0.15 pg/l. All measurements were above this limit. All samples for each individual were analyzed twice. The mean coefficient of variation, CV, between the two samples were 7.91%. All samples with a mean CV exceeding 20% were rerun. In total 11 samples were rerun, and all those samples were within acceptable CV-ranges after a second run, and could be used for final analysis.

### 2.6 Kynurenic and quinolinic acid analysis

Plasma concentrations of KYNA and QUIN were quantified using ultra performance liquid chromatography – tandem mass spectrometry as described earlier (Trepci et al., 2020). The metabolites were detected in higher concentrations than the lowest level of quantification in all samples. Six samples were run in duplicates and the mean coefficient of variation was less than 5%.

### 2.7 Statistical analysis

Participant characteristics are presented in Table 1 with p-values derived from Kruskal-Wallis rank sum tests for continuous variables and Pearson's Chi-squared test for dichotomous variables.

Linear mixed models (LMM) were used to explore the effects of CM, SUD and time on the outcomes of interest: IL-6, KYNA as well as ratio of KYNA and QUIN. A first order autoregressive covariance pattern and Satterthwaite correction was employed for all LMMs.

Since IL-6 was right-skewed, data was first log-transformed, with 10 as a base. Participants were then grouped into CM vs. no CM; and SUD vs. no SUD. Baseline was defined as 15 min prior to initiation of the stress test, i.e. before any test was performed. Timepoint was included as a factor in all analyses. For IL-6, we first fitted a full model with a two-level grouping variable, CM/no CM and SUD/no SUD, with time as a categorical variable with 5 levels, and group by time interactions in the LMM. In the next step we fitted a second model using a four-level grouping variable: CM only, CM + SUD, SUD only, and HC and time as a categorical variable. Pairwise comparisons were conducted for the 4-level grouping variable.

For KYNA and the ratio of KYNA/QUIN we also started with the same level grouping variable. In the next step we continued with a second model using the same four groupings as previously: CM only, CM + SUD, SUD only and HC, with time as a categorical variable. In sensitivity analyses, we also controlled for BMI, when conducting the analysis on CM/no CM, and SUD/no SUD. It was not deemed possible to conduct similar analyses on the model with 4 groups, due to lack of power.

The stress-data was significantly skewed to the right, and was therefore log-transformed with 10 as a base. A linear mixed model was then conducted to assess differences of self-reported stress between groups, looking both at two- and four-grouping variables. Correlations between perceived stress and IL-6 and KYNA/QUIN-ratio were conducted using a linear mixed model, with IL-6 and KYNA/QUIN-ratio as the dependent variable and stress as the fixed effect.

Descriptive statistics were obtained using RStudio (version 4.3.2) (RStudio Team, 2020), LMM analyses were conducted using SPSS, version 29.0.2 (IBM, 2020).

## 3 Results

### 3.1 Participant characteristics

Groups were sex and age matched with no significant differences between groups. Both past and present psychiatric disorders were more common in the CM only, CM+SUD and the SUD only groups compared to the HC group (table S1). The use of psychotropic medications also differed significantly across groups (p = 0.003). While the majority in the CM+SUD and the SUD only group (56%) used at least one psychotropic medication, only 8.3% of the HC group had any psychotropic medication. Most common psychotropic medications used were antidepressants (SSRI or SNRI). As expected, AUDIT (p = 0.033) and DUDIT (p < 0.001) scores were significantly higher in the group with lifetime SUD; as was CTQ-scores high in the group exposed to childhood trauma (p < 0.001) (Table 1).

### 3.2 IL-6

**Fig. 1:**
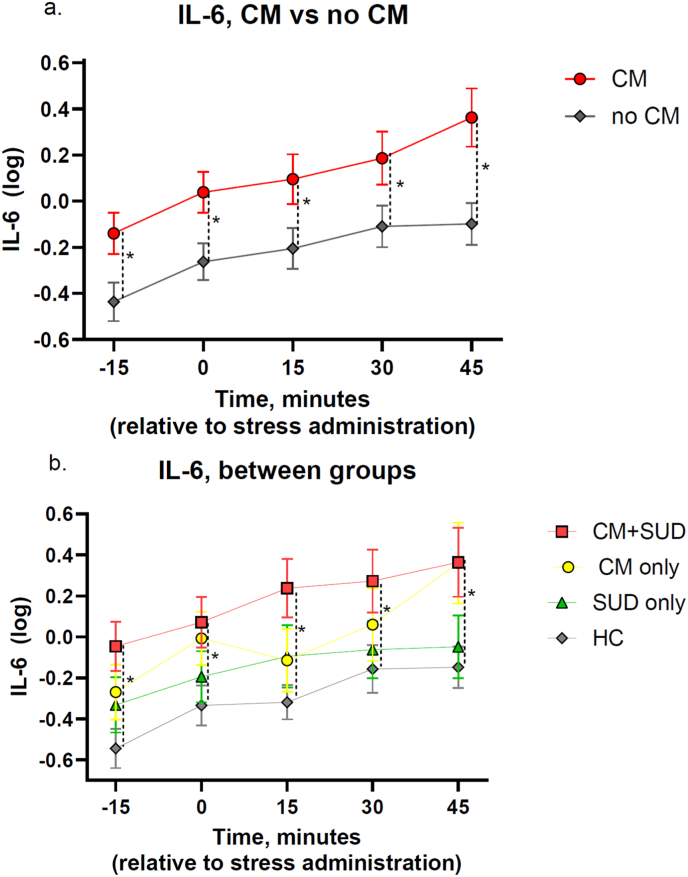
**Effect of acute stress on IL-6 concentrations by childhood maltreatment (CM) and lifetime substance use disorder (SUD) status. a.** There was a significant difference in IL-6 concentrations between participants exposed to CM, and those who had not (p = 0.008) **b.** There was an increase in IL-6 concentrations over time, for all participants (p < 0.001). There was a significant difference in IL-6 concentrations between the CM + SUD-group compared to the HC-group (p = 0.024).

Participants exposed to CM exhibited significantly higher IL-6 concentrations, overall, compared to those without CM exposure, irrespective of their SUD status (mean difference = 0.327, 95% CI = 0.087-0.567, p = 0.008; Table 2a, Fig. 1a), this correlation remained statistically significant, after controlling for BMI (p = 0.016, Table S3). Notably, participants with CM + SUD had significantly higher IL-6 concentrations compared to healthy controls, also after adjusting for multiple comparisons (mean difference = 0.490, 95% CI = 0.04-0.94, p = 0.024; Fig. 1b–Table 2a). Conversely, there was no significant difference in IL-6 concentrations between participants with and without SUD (p = 0.114, Table 2a).

**Table 2a:** Linear mixed model showed a significant difference in IL-6 levels between those exposed to CM and those not exposed (p = 0.008). Post-hoc analysis revealed a significant difference in IL-6 levels between the CM + SUD-group and HC (p = 0.024). CM = Childhood maltreatment. SUD = Substance use disorder. HC = Healthy controls. KYNA = Kynurenic acid. QUIN = Quinolinic acid.

2a. IL-6					
Group comparison	Mean Diff	SE	df	p-value	95% CI
CM vs no CM	0,327	0,121	77,12	**0,008∗**	−0,567 to −0,087
SUD vs no SUD	0,2	0,125	77,32	0,114	−0,448 to 0,049

**Post-hoc test - IL-6**					
CM vs CM + SUD	−0,2	0,176	75,86	0,835	−0,676 to 0,276
CM vs HC	0,29	0,180	75,67	0,506	−0,196 to 0,776
CM vs SUD	0,133	0,178	75,85	0,974	−0,347 to 0,614
CM + SUD vs HC	0,49	0,165	74,86	**0,024∗**	0,044 to 0,963
CM + SUD vs SUD	0,334	0,163	75,05	0,237	−0,107 to 0,774
HC vs SUD	−0,156	0,167	74,88	0,926	−0,607 to 0,295

After experimental acute stress exposure, IL-6 concentrations showed a significant increase in all participants (p < 0.001, Table S3). However, there was no significant interaction between CM and time (p = 0.18, Table S3) or between SUD and time (p = 0.227, Table S3), indicating that the changes in IL-6 concentrations over time were not significantly influenced by the presence of CM or SUD.

### 3.3 KYNA

There was no main effect of CM on KYNA-levels (mean difference = 0.001, CI = −0.01-0.01, p = 0.744, Table S2), or SUD (mean difference = 0.004, CI = −0.01-0.01, p = 0.10, Table S2), nor was there an interaction effect between CM and time (p = 0.803) or between SUD and time (p = 0.166). This indicates that changes in KYNA concentrations over time were not significantly influenced by CM or SUD.

### 3.4 KYNA/QUIN-ratio

**Fig. 2:**
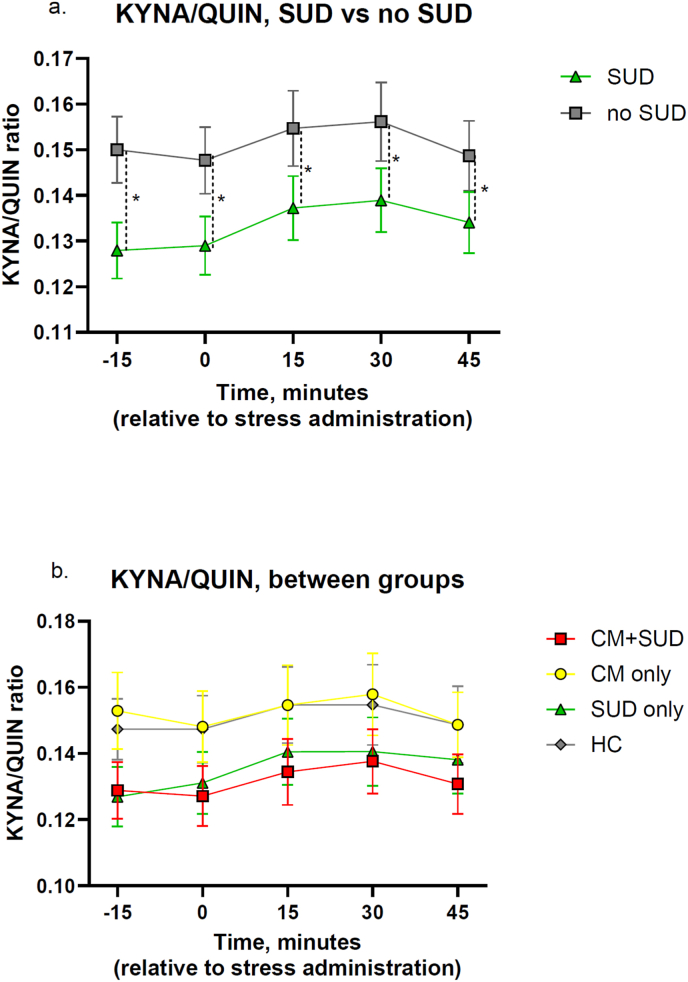
**Effects of stress on KYNA/QUIN concentrations by childhood maltreatment (CM) and lifetime substance use disorder (SUD) status.** There was no effect over time on KYNA/QUIN. a. There was a significant decrease in KYNA/QUIN-ratio in participants with ongoing, or a history of, substance use disorder, compared to those without (p = 0.047). b. Post-hoc test did not reveal any significant difference between groups of KYNA/QUIN-ratio.

There was no significant difference in the KYNA/QUIN ratio between participants with and without CM (mean difference = 0.001, CI = −0.02-0.02, p = 0.925, Table 2b). However, participants with SUD showed a significantly decreased KYNA/QUIN ratio (mean difference: -0.02, 95% CI: 0.00-0.04, p = 0.047 Fig. 2a, Table 2b), suggesting an association between SUD and a lower KYNA/QUIN ratio. This association remained significant after controlling for BMI (p = 0.036, Table S3). There were no significant interactions between CM and time (p = 0.67, Table S3) or between SUD and time (p = 0.58, Table S3), indicating that changes in the KYNA/QUIN ratio over time were not significantly influenced by CM or SUD.

**Table 2b:** There was a significant difference in KYNA/QUIN ratio between participants with ongoing or previous SUD compared to those not exposed (p = 0.047). There was no significant difference between groups in post-hoc analysis. CM = Childhood maltreatment. SUD = Substance use disorder. HC = Healthy controls. KYNA = Kynurenic acid. QUIN = Quinolinic acid.

2b. KYNA/QUIN-ratio					
Group I	Mean Diff	Std Error	DF	p-value	95% CI
CM vs no CM	0,001	0,010	85,749	0,925	−0,021 to 0,019
SUD vs no SUD	−0,02	0,010	85,83	**0,047∗**	0,00 to 0,039

**Post-hoc test - KYNA/QUIN**					
CM vs CM + SUD	0,024	0,014	83,96	0,425	−0,014 to 0,062
CM vs HC	0,007	0,014	84,16	0,998	−0,031 to 0,045
CM vs SUD	0,022	0,015	84,23	0,561	−0,017 to 0,062
CM + SUD vs HC	−0,017	0,014	83,55	0,740	−0,054 to 0,019
CM + SUD vs SUD	−0,002	0,014	83,65	1,000	−0,039 to 0,035
HC vs SUD	0,015	0,014	83,86	0,852	−0,022 to 0,053

### 3.5 Self-reported stress

Self-reported stress, assessed with a 100 mm visual analog scale, had a mean value of 83, with a standard deviation of 16.5 (Table S4a). There was a robust effect of stress over time (p < 0.001) for all participants. There were no significant differences between groups in acute stress-response: either for CM (p = 0.083) SUD-status (0.199) or four grouping variables (p = 0.114) (Table S4b). There was no significant correlation between self-reported stress-response and IL-6 levels (p = 0.834) or KYNA/QUIN-ratio (p = 0.255) (Table S4b).

## 4 Discussion

We explored the association between prospectively documented CM and a lifetime history of SUD, on IL-6 and kynurenine metabolites. As hypothesized, we found increased levels of the proinflammatory biomarker IL-6 in participants exposed to prospectively documented CM. Contrary to our hypothesis, we found no association between CM exposure and kynurenine metabolite levels. Altered kynurenine metabolite levels were however associated with life-time history of SUD. Thus, histories of CM and SUD had individual effects on proinflammatory signaling and kynurenine metabolism, respectively, pointing to potential novel therapeutic opportunities in these populations.

Most research regarding CM is based on retrospectively reported maltreatment (Grummitt et al., 2024; Daníelsdóttir et al., 2024). A recent meta-analysis has shown a difference in association between retrospective and prospective measures of CM and psychopathology (Baldwin et al., 2024). This might imply that first-person, subjective memory-appraisal of maltreatment (retrospective measure) is a factor in the association of CM and psychopathology, compared to third-person accounts of maltreatment (prospective measures). By assessing prospective CM and its relationship to inflammation, kynurenine metabolites, and SUD later in life, this study adds to the limited research on individuals with prospective CM exposure.

In the present study, we examined both chronic effects of severe early-life stress (CM) and its consequences for acute stress reactivity using an established experimental stress paradigm. Chronic and acute stress are closely linked: the allostatic load hypothesis posits that negative consequences of repeated acute stress responses accumulate over time, resulting in long-term physiological adaptations, including low-grade inflammation (McEwen and Eliot, 1993). Experimental evidence further supports this connection, showing associations between acute stress responses (e.g., cortisol reactivity) and markers of chronic stress, such as hippocampal volume (Degering et al., 2023). Despite this established relationship, the mechanisms through which repeated acute stress responses translate into persistent biological changes remain poorly understood (Rohleder, 2019) including the threshold at which acute stress becomes chronically dysregulating (Renner et al., 2022) In this context, our finding that participants exposed to CM had significantly elevated baseline IL-6 levels is consistent with previous research indicating that early life stressors, such as CM, can lead to long-lasting alterations in immune system functioning and elevated inflammatory markers such as IL-6 (Baumeister et al., 2016; Powell et al., 2013). The MAST-task elicited a reliable stress response in all participants, in accordance with prior research (Smeets et al., 2012). However, since the test combines a physical and a psychosocial stressor, it does not allow changes in the response to the task to be specifically linked to one of these two stressor categories. The MAST elicited a robust subjective stress response, with no difference between groups, with mean ratings approaching the maximum value of 100 and a pronounced right-skewed distribution. This high overall stress response may limit the interpretability of group differences or associations with IL-6 and KYNA/QUIN ratio. Although no significant group differences or biomarker associations were observed, this may reflect a ceiling effect, whereby uniformly elevated stress ratings obscure potential variability between groups. Future studies may benefit from using more sensitive stress measures to better capture individual differences in subjective stress responses and their associations with CM exposure, SUD, inflammatory reactivity, and kynurenine metabolism.

What is novel in our study is the impact of childhood maltreatment that was assessed prospectively, rather than retrospectively. A limitation of previous findings was that they were mainly based on retrospectively self-reported CM, which only shows modest agreement with prospective assessment (Baumeister et al., 2016; Baldwin et al., 2019; Reuben et al., 2016; Löfberg et al., 2023). Our data are therefore important, in that they are based on prospectively recorded CM, and therefore support that an association between CM and proinflammatory activation remains even when the bias potentially inherent to retrospective self-report is eliminated. Our study also showed that participants with both CM and SUD had the highest IL-6 concentrations, suggesting a compounded effect of CM and lifetime SUD on proinflammatory activity.

We found a robust main effect of increased IL-6 over time in response to the stressor, irrespective of CM or SUD status. This replicates previous findings and highlights a possible mechanism linking psychological stressors to physiological changes implicated in psychiatric disorders (Renner et al., 2022). However, IL-6 responses to the stress challenge did not differ as a function of CM or SUD status. This contrasts with previous studies, which found increased IL-6 reactivity to stress in individuals with a self-reported history of CM (Marsland et al., 2017; Pace et al., 2006). A possible reason for this discrepancy may be the timing of IL-6 measurement. We measured IL-6 up to 45 min post-stress, whereas other studies measured IL-6 reactivity at later time points (Pace et al., 2006; Brooks et al., 2020). IL-6 concentrations may not have peaked at 45 min when our measurements ended, and we thus may have missed part of the IL-6 increase (Marsland et al., 2017). Another potential reason for the difference between findings is that we used prospectively established history of CM exposure in our study. Retrospective self-report of CM history, used in earlier research, has been shown to largely reflect ongoing, primarily internalizing psychopathology, likely associated with proinflammatory activation (Reuben et al., 2016). Thus, both our assessment of CM (prospective vs retrospective) and blood sample timing are plausible factors impacting the discrepancy with existing literature, though we cannot distinguish between these factors in the current dataset.

Participants with SUD had a lower KYNA/QUIN ratio compared to those without SUD, implying a possible association of the kynurenine pathway to SUD. This finding suggests that SUD might be associated with a shift towards a more neurotoxic kynurenine pathway metabolism, potentially contributing to the pathophysiology of SUD. Similar findings have previously been reported in patients with AUD (Leclercq et al., 2021). Lower KYNA/QUIN ratio supports the hypothesis that SUD is linked to an imbalance in neuroactive kynurenine metabolites, potentially increasing glutamatergic activity, and resulting dopaminergic dysfunction. Future work exploring whether this metabolic change is a consequence or antecedent of SUD development would be valuable in elucidating this relationship.

This is the first study to measure kynurenine metabolites in people exposed to CM. While previous studies conducted on animals support an association between early life adversity and alterations in kynurenine metabolism (Coplan et al., 2021)), we did not find that these effects translate to humans. Also, we did not replicate previous findings regarding an increase of KYNA in response to psychosocial stress (La Torre et al., 2021). While this latter study was conducted in healthy males, our sample included both males and females, and participants exposed to CM exposed and life-time SUD which potentially could explain the discrepancy in results.

The kynurenine pathway is influenced by proinflammatory cytokines, but our study did not find any significant associations between IL-6 concentrations and kynurenine metabolites. The IL-6 response was dynamic, while kynurenine metabolites remained relatively stable over time, and were not measurably impacted by a stressor that was sufficient to produce a robust activation of the HPA axis (Perini et al., 2023). This raises the possibility that SUD induced lasting changes in kynurenine metabolism, potentially steering it towards its more neurotoxic/glutamatergic pathway. Whether this is associated with proinflammatory or other processes is unclear. To assess these associations, future prospective studies should assess the long-term effects of proinflammatory activity and of substance use on kynurenine metabolism.

The study has two main limitations. First, the sample sizes afford limited power to assess the independent and interactive effects of CM and SUD. The sample size also limited the possibility to control for potential covariates. We conducted one sensitivity analysis, in which we controlled BMI. Controlling for psychotropic medication was not deemed possible, due to the variability in psychotropic medications used in the samples and the increased risk of type II error, given the sample size. The most common psychotropic medications, however, were antidepressants. Since antidepressants have a known anti-inflammatory effect (Strawbridge et al., 2015), it seems unlikely that the increase in IL-6 in the CM-cohort would be due to medication. Second, sampling of biomarkers was only for 45 min following the stress challenge, while the expected peak of IL-6 concentrations is at 60-90 min post-stress (Marsland et al., 2017; Brooks et al., 2020). We therefore cannot exclude that a longer sampling duration would have identified differences in the dynamics of the IL-6 response to acute stress as a function of CM, SUD or both.

In summary, these results contribute to a growing body of evidence indicating that CM can lead to chronic immune system dysregulation that can be indexed using elevated IL-6 levels as a biomarker. This chronic proinflammatory activation may contribute to the elevated risk of developing psychiatric disorders, including SUD, in people with CM exposure. Our findings also support the notion that SUD is associated with alterations in the kynurenine pathway, and specifically a shift towards neurotoxic metabolites. This could exacerbate neuroinflammatory processes and contribute to the persistence of psychiatric symptoms.

## Funding

This study was funded by the Swedish Research Council 2013–2024, funding recipient Markus Heilig, grant nos. 2013–07434; the Medical Training and Research Agreement in Ostergotland Region, grant no. ALF 2017: LIO-599451 main funding recipient Per Gustafsson; 10.13039/100023254ALF 2018: LIO-692621, and ALF 2019: LIO-791581, ALF 2020: RO−888021; and ALF 2021: RO−935602 main funding recipient Andrea J Capusan; by Fonden för Psykisk Hälsa, Stiftelsen Frimurare Barnhuset Stockholm to Elisabeth R Paul; by Systembolagets alkoholforskningsråd, grant numbers: 2016–0018, 2017–0075, 2018–0030, and 2019–0007 main recipient Markus Heilig; and by the 10.13039/100000874Brain & Behavior Research Foundation NARSAD Young Investigator Grant 27094 to Leah M Mayo.

## CRediT authorship contribution statement

**Lars Östman:** Conceptualization, Data curation, Formal analysis, Software, Validation, Writing – original draft, Writing – review & editing. **Elisabeth R. Paul:** Conceptualization, Data curation, Formal analysis, Software, Supervision, Writing – original draft. **He Zhang:** Data curation, Formal analysis, Writing – original draft. **Evelina Larsson:** Data curation, Formal analysis, Project administration. **Irene Perini:** Conceptualization, Data curation, Investigation, Methodology, Project administration, Supervision. **Lilly Schwieler:** Data curation, Resources. **Sophie Erhardt:** Data curation, Resources. **Lovisa Holm:** Data curation, Formal analysis. **Åsa Axén:** Data curation, Resources. **Leah M. Mayo:** Conceptualization, Funding acquisition, Project administration, Resources, Supervision, Writing – review & editing. **Markus Heilig:** Conceptualization, Formal analysis, Investigation, Methodology, Project administration, Resources, Supervision, Writing – original draft, Writing – review & editing. **Andrea J. Capusan:** Conceptualization, Data curation, Formal analysis, Funding acquisition, Investigation, Methodology, Project administration, Supervision, Validation, Writing – original draft, Writing – review & editing.

## Declaration of competing interest

Markus Heilig has received speaker's fees, research funding and/or scientific advisory board compensation from Lundbeck, Aelis Farma, Indivior, Brainsway Technologies, Accord Pharma, Nordic Drugs and Janssen Pharmaceuticals, all outside the scope of current project. Andrea Johansson Capusan has received speaker's fees, and/or scientific advisory board compensation from Indivior, Camurus, dnepharma, Nordic Drugs all outside the scope of the current project. Leah M. Mayo has received consultation compensation from Synendos Therapeutics, which is unrelated to the current manuscript.

## Data Availability

The authors do not have permission to share data.
